# A model of pulldown alignments from SssI-treated DNA improves DNA methylation prediction

**DOI:** 10.1186/s12859-019-3011-2

**Published:** 2019-08-19

**Authors:** Blythe S. Moreland, Kenji M. Oman, Ralf Bundschuh

**Affiliations:** 1 Department of Physics, The Ohio State University, Columbus, OH USA; 2 Fred Hutchinson Cancer Research Center, Seattle, WA USA; 3 Department of Chemistry&Biochemistry, Division of Hematology, and Center for RNA Biology, The Ohio State University, Columbus, OH USA; 4 Present address: Institute for Genomic Medicine, Nationwide Children’s Hospital, Columbus, OH, USA

**Keywords:** DNA methylation, Protein pulldown, Methyl-CpG-binding domain proteins, SssI treatment

## Abstract

**Background:**

Protein pulldown using Methyl-CpG binding domain (MBD) proteins followed by high-throughput sequencing is a common method to determine DNA methylation. Algorithms have been developed to estimate absolute methylation level from read coverage generated by affinity enrichment-based techniques, but the most accurate one for MBD-seq data requires additional data from an SssI-treated Control experiment.

**Results:**

Using our previous characterizations of Methyl-CpG/MBD2 binding in the context of an MBD pulldown experiment, we build a model of expected MBD pulldown reads as drawn from SssI-treated DNA. We use the program BayMeth to evaluate the effectiveness of this model by substituting calculated SssI Control data for the observed SssI Control data. By comparing methylation predictions against those from an RRBS data set, we find that BayMeth run with our modeled SssI Control data performs better than BayMeth run with observed SssI Control data, on both 100 bp and 10 bp windows. Adapting the model to an external data set solely by changing the average fragment length, our calculated data still informs the BayMeth program to a similar level as observed data in predicting methylation state on a pulldown data set with matching WGBS estimates.

**Conclusion:**

In both internal and external MBD pulldown data sets tested in this study, BayMeth used with our modeled pulldown coverage performs better than BayMeth run without the inclusion of any estimate of SssI Control pulldown, and is comparable to – and in some cases better than – using observed SssI Control data with the BayMeth program. Thus, our MBD pulldown alignment model can improve methylation predictions without the need to perform additional control experiments.

**Electronic supplementary material:**

The online version of this article (10.1186/s12859-019-3011-2) contains supplementary material, which is available to authorized users.

## Background

Affinity enrichment-based techniques for methylated DNA capture remain a cost-effective method for achieving genome-wide coverage of the CpG methylome [1–3]. Antibodies may be used to bind specifically to denatured methylated DNA (methylated DNA immuno-precipitation, MeDIP-seq [4]), or the binding domain of Methyl-CpG-binding domain (MBD) proteins may be used to bind specifically to double-stranded methylated CpGs (MBD-seq [5]). Through incubation and pulldown with one of these types of agents, DNA enriched for methylation is captured and then sequenced in a high-throughput manner, reducing sequencing costs while still mapping ∼70*%* and ∼80*%* of all mCpGs in the human genome for MeDIP-seq and MBD-seq, respectively [1, 6]. These methods have been used to identify patterns of methylation associated with gene expression and cell phenotypes, for instance MBD-seq in the methylome profiling of cancer [7–10].

Since pulldown reads are sequenced and aligned without knowing which of the CpGs on the DNA fragment were methylated, MBD-seq data are often processed around the resolution of the DNA fragment length, typically in 100-500 bp windows. The interpretation of MBD pulldown reads is also affected by the density and arrangement of mCpGs on the fragment, which is known to influence the efficiency of capture by MBD pulldown [11–13]. Thus, statistical approaches must be used to quantify methylation levels from MBD pulldown alignments and to increase its resolution to make it competitive with bisulfite sequencing techniques. These bisulfite sequencing techniques — whole genome bisulfite sequencing (WGBS) and reduced representation bisulfite sequencing (RRBS) — remain the gold standard of methylation prediction. However, they are still held back by sequencing and data processing costs (in the case of WGBS) and restrictions in genome coverage (in the case of RRBS). Hence the optimization of MBD pulldown analysis is still important to methylome epigenetics, especially for exploratory studies with large numbers of samples.

**Fig. 1:**
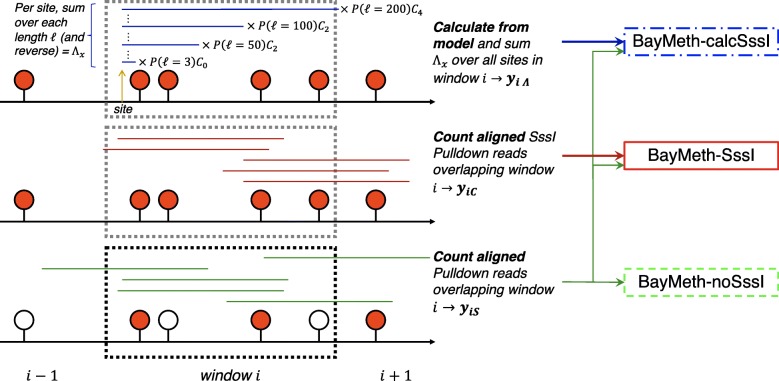
Inputs for running BayMeth. On the left, obtaining read coverage of a genomic window *i* with some CpG pattern (circles) where the CpG is either methylated (red) or unmethylated (empty). For the experimentally-derived inputs this is done by counting the number of aligned reads that overlap the window from an MBD pulldown experiment done on a sample of interest (*y*_*iS*_=5 for the window depicted on the bottom) or on an SssI-treated sample (*y*_*iC*_=5, for the window depicted in the middle). With our implementation of a calculated SssI Control proxy, we incorporate the range of fragment lengths (*ℓ*, where *P*(*ℓ*) is the probability a fragment of length *ℓ* is in the library) and the amount of SssI pulldown expected (*C*_*n*_) for a fragment given the number of accessible mCpGs (*n*) on the fragment. For a given site, *x*, within our window *i*, we calculate a term *Λ*_*x*_ that sums over *P*(*ℓ*)*C*_*n*_ terms for all fragments that begin in the window (on the forward or reverse strands), and then sum over all these *Λ*_*x*_ values in the window to calculate *y*_*iΛ*_. On the right, arrows indicate which quantities are used as inputs into each BayMeth mode considered

Various algorithms have been used to quantify absolute methylation levels, or determine differentially methylated regions directly from read counts, for both MBD-seq [14–16] and MeDIP-seq [17–20] data. The program BayMeth has shown the highest accuracy in predicting methylation from MBD pulldown coverage, as determined by comparison to methylation levels calculated by WGBS [14]. Specifically, BayMeth performs best when control data from MBD pulldown run on a fully-methylated control sample are available (Fig. 1). To generate such a sample, DNA is treated with SssI CpG methyltransferase, which methylates Cs in the CpG dinucleotide context [21], and thus pulldown from this sample can inform the expected number of reads from that genomic region at 100% methylation. BayMeth then uses an empirical Bayes approach to model expected MBD pulldown read densities conditioned on the level of methylation and the CpG density of the region.

Given our previous characterizations of methylated DNA and MBD2 interactions [13], we built a model of MBD pulldown alignments from SssI-treated DNA that we tested the efficacy of through substitution for the SssI control data set utilized by the BayMeth model. Our model incorporates the fragment length distribution in the MBD pulldown library, the minimum separation between neighboring mCpGs needed for optimal pulldown efficiency, and the relative representation of DNA fragments with *n* mCpGs to those with 0 mCpGs, and generates an expected MBD pulldown for every site in the human genome from SssI-treated DNA (Fig. 1). We find high correlation between the calculated pulldown coverage, generated from our model of MBD pulldown alignments, and observed pulldown coverage from an SssI-treated control. Using our modeled pulldown coverage in conjunction with the BayMeth program produces methylation predictions that are comparable to those produced by BayMeth using observed control data, and in some cases predictions using our model are better. Additionally, in all cases tested in this study, BayMeth used with our modeled pulldown coverage performs better than BayMeth run without the inclusion of any estimation of SssI control pulldown. This shows that our MBD pulldown alignment model can improve methylation predictions without the need to perform additional control experiments. Source code implementing our model can be found at http://bioserv.mps.ohio-state.edu/SssICalc.

## Methods

### MBD pulldown experiment and methylation reference

Pulldown data for a Sample of Interest were taken from [22] to evaluate methods of methylation quantification. These pulldown experiments were done using the MethylMiner kit, which uses a biotinylated form of the protein MBD2 to capture highly methylated fragments. In addition, single CpG methylation fraction derived from RRBS is used from that study to verify predictions. Bisulfite treatment converts an unmethylated C to a U, thus a sequenced T/A aligning to an encoded C/G (depending on the strand) in the CpG context indicates absence of methylation. To calculate RRBS methylation fraction for a genomic window *i* that contains CpGs indexed by *j*, let *r*_*j*_ represent the number of RRBS reads that overlap CpG *j*, and *m*_*j*_ the number of those reads that overlap and read as not bisulfite converted at the position of CpG *j* (i.e. CpG *j* is methylated). Then $\mu _{i}^{\text {RRBS}}$, the RRBS methylation level, is $\frac {\sum \nolimits _{j \in i} m_{j}}{\sum \nolimits _{j \in i} r_{j}}$.

### Modeled pulldown from SssI-treated DNA

Pulldown data is analyzed per genomic window *i*. We use the construction *x*∈*i* to refer to all genomic positions *x* that fall within genomic window *i*. Our model for *Λ*_*x*_, the expected pulldown at position *x*, can be used to calculate MBD pulldown signal from window *i* by summing over all *Λ*_*x*_ terms in the window and rounding down to the nearest integer, $y_{i\Lambda } = \left \lfloor \sum \nolimits _{x \in i} \Lambda _{x} \right \rfloor $, so that *y*_*iΛ*_ can represent a physical read count like the window coverage inputs that BayMeth takes (see Fig. 1 and “BayMeth implementation” subsection).

Three ingredients are used to calculate the expected pulldown of a particular fragment of length *ℓ* to a location *x*: (i) The number of accessible mCpGs on the fragment, (ii) the relative enrichment of that fragment due to this number of mCpGs, and (iii) the probability of a fragment of length *ℓ* being sequenced in the pulldown library. Ingredient (i) depends on the minimum separation of consecutive mCpGs in order for the two to be bound by two separate MBD2 domains, set here to be 3 bp [13]. Ingredient (ii) depends on the pulldown efficiencies as a function of accessible CpGs. These pulldown efficiencies were calculated in [13] from the same MethylMiner kit as used here, and we thus use the pulldown efficiencies *E*(*n* mCpGs) for *n* well-separated CpGs from [13]. Then, the coefficients for (ii) are derived as *C*_*n*_=*E*(*n* mCpGs)/*E*(0 mCpGs) (Table 1). We set the maximum number of mCpGs considered here to 7, which aligns with the MethylMiner Kit estimate. To derive a sample-to-sample standard deviation, *s*_*ss*_, we sub-sampled the fragments by chromosome, and calculated *C*_*n*_ on each sub-sample (Table 1 and Additional file 1: Table S1). These standard deviations are under 5% except for *C*_6_ and *C*_7_ due to the relative rarity of fragments with 6 or more CpGs that satisfy our criteria for analysis. While the standard deviation for *C*_7_ is particularly large, given the overall depth of the input data, we were previously able to show that for *n*≤7,*E*(*n* mCpGs) differs to a statistically significant degree when one calculates it with the subset of fragments containing only “well-separated” CpGs versus allowing any separation. As the alternative would be to set *C*_7_ to *C*_6_, we propagate the modest increase allowed by setting *C*_7_≈ 260. The fragment length distribution required for (iii) has not been derived before and will be discussed in more detail in a later section. Source code for calculating *y*_*iΛ*_ and example data files from hg18 and hg19 reference genomes can be found at http://bioserv.mps.ohio-state.edu/SssICalc.

**Table 1:** MBD Pulldown scale factors

*n* mCpGs	0	1	2	3	4	5	6	≥7
*C*_*n*_	1	1.489	5.468	32.31	124.7	207.3	233.2	259.7
*%* *s*_*ss*_	-	1.7%	2.3%	2.2%	3.3%	4.8%	19%	81%

These scale factors are ratios of pulldown efficiencies comparing the SssI Control and Input Control libraries, and represent the probability that a fragment with *n* mCpGs will be pulled-down, sequenced, and aligned relative to a fragment with 0 mCpGs. A sample-to-sample standard deviation, *s*_*s*_*s*, was calculated by sub-sampling by chromosome, and is given as a percentage of the *C*_*n*_ value

### BayMeth implementation

The BayMeth algorithm [14] was run using pulldown read coverage calculated per genomic window *i* from just a Sample of interest (*y*_*iS*_), with additional pulldown read coverage data from an SssI-treated Control sample (*y*_*iC*_), or with calculated control data generated from our model of pulldown from SssI-treated DNA, *y*_*iΛ*_, used in place of *y*_*iC*_. These inputs define the three implementations of BayMeth that we consider, which we call BayMeth-noSssI, BayMeth-SssI, and BayMeth-calcSssI respectively (Fig. 1). We use the default parameters and recommended prior distributions for calculating the normalization offset *f* and hyperparameters *α* and *β*. For calculating a local CpG density for each genomic window, we include bases within an average fragment length of the window range (to set the window parameter for the cpgDensityCalc function in the Repitools package). To calculate read counts *y*_*iS*_ and *y*_*iC*_, the length of each fragment is approximated by the average fragment length, and then for each genomic window the number of reads that overlap the window is counted.

### Methylation quantification evaluation

Methylation fraction estimates on our Sample of interest were calculated on non-overlapping, fixed-width windows covering hg18. We use windows of 100 bp (as in the original BayMeth paper) and 10 bp. BayMeth-SssI, BayMeth-noSssI, and BayMeth-calcSssI were evaluated on genomic windows with RRBS coverage of 10 or more and at least 75% mappable bases. To determine mappability, we use project ENCODE’s mappability calculation for each 36mer in the hg18 genome [23], which allows for no more than 2 mismatches. Then the mappability of the window is the fraction of bases with scores of 1. We also compare methylation quantifications on the Sample of interest in the original BayMeth publication [14] using their selection criteria of 100 bp windows on chromosome 7 with at least 33 WGBS read coverage and at least 75% mappable bases as determined by unique Bowtie alignment.

To quantify performance of each methylation estimate method, we calculate Receiver Operator Characteristic (ROC) curves. For the ROC curve, the true methylation state of each considered window is determined to be “methylated” if the BS methylation estimate on that window $\mu _{i}^{\text {RRBS}} > 0.50$ and “unmethylated” if $\mu _{i}^{\text {RRBS}} \leq 0.50$. Each estimate method produces a set of predicted methylation levels {*μ*_*i*_}. By sorting this list and varying the cutoff that splits the “methylated” and “unmethylated” groups, each point on the ROC curves represents a split that is evaluated by calculating the resulting True positive rate and False positive rate. The area under the ROC curve (AUC) is thus a measure of how well the method’s predictions serve as an indicator of methylation state, the maximum value being 1.

## Results

### A model of pulldown data from SssI-treated DNA

An MBD enrichment-capture experiment produces a pool of DNA fragments enriched for DNA methylation. Those fragments are sequenced and the reads are aligned to a reference genome to form the “pulldown” data set. In order to generate a control sample that approximates the pulldown of a genomic window at full methylation, the experiment can be done on DNA treated with M.SssI to methylate all cytosines in the CpG dinucleotide context. In this study, we formulate a model of the expected pulldown data from such an SssI Control sample to use in place of an experimental SssI Control pulldown data set. Let *Λ*_*x*_ represent the average number of pulldown reads that align to the genome starting at location *x*. For our purposes, we assume that a fragment that aligns to location *x*, on the forward or reverse strand, with a length *ℓ* possesses the corresponding sequence that is encoded in the genome. From our previous results [13], the parameters that most determine the probability that such a fragment starting at *x* would be pulled down are the number and spacing of the CpGs on the DNA fragment. We summarize the number and spacing of the CpGs by our term “accessible CpGs”. This refers to the number of CpGs on the fragment that can be simultaneously bound by MDB2 protein domains after taking into account that MBD2 domains are sterically excluded from binding if the CpGs are too close to each other along the DNA molecule. Thus, for *Λ*_*x*_, we consider every fragment that could align starting at *x* and sum over the expected amount of pulldown of that fragment, weighted by the probability that a fragment of that length would appear in the sample to begin with:

1 $$ \Lambda_{x} \propto \sum\limits_{\ell = \ell_{\min}}^{\ell_{\max}} P(\ell) [C_{n(x \to x+\ell-1)} + C_{n(x-\ell+1 \to x)}],   $$

where the sum is over the range of possible pulldown fragment lengths *ℓ*. In Eq. (1), *P*(*ℓ*) represents the probability that a fragment sequenced from the pulldown data set is of length *ℓ*,*n*(*a*→*b*) the number of accessible CpGs that would be on a fragment that starts at genomic location *a* and ends at *b* (inclusive), and *C*_*n*_ the scale factor for the representation of sequenced fragments that have *n* accessible mCpGs. The two terms correspond to alignments on the forward strand and reverse strand that could both align to location *x*. These *C*_*n*_ factors scale the probability of observing a fragment with *n* accessible mCpGs to that of observing a fragment with 0 mCpGs. The other normalization to consider is that which scales *Λ*_*x*_ to the sequencing depth of the modeled experiment. We choose a pre-factor of 1 and later show that the overall results are not affected by this choice over a large range of values.

To evaluate Eq. (1) for each site in a reference genome, parameters *n*(*a*→*b*),*C*_*n*_, and *P*(*ℓ*) were derived as detailed next from the experiments performed in [13]. These experiments resulted in two data sets. For both data sets the DNA was first treated with SssI and then fragmented. For the Input Control (*I*) data set, the DNA fragments were simply sequenced. The SssI Control (*C*) data set, on the other hand, represents the library of DNA fragments that were submitted to the enrichment-capture experiment, pulled down, and then sequenced. Comparing SssI Control and Input Control data sets yields the parameters of our model as follows:

#### Number of accessible CpGs

In [13], we deduced that the physical size of the binding protein used for the pulldown experiment (in this case MBD2) can limit the accessibility of an mCpG to a binding protein if another nearby mCpG has already bound a protein. Specifically, we found that pulldown efficiency was suppressed for fragments with two mCpGs separated by 2 bp or less, relative to those with separations ≥3 bp. We want the number of “accessible CpGs” to refer to the largest number of CpGs that can be simultaneously bound by protein. To approximate this, we calculate *n*(*x*→*x*+*ℓ*−1) for the DNA sequence represented by [*x,x*+*ℓ*−1], by finding the largest subset of CpGs on the sequence such that each pair of CpGs in the subset are separated by at least 3 bp. This is equivalent to allowing an MBD protein to be bound at the first CpG, where the location of the CpG is identified with the position of the Cytosine and labeled *c*_1_. Then, going through the rest of the downstream CpGs, the number of bound CpGs is only increased, and the location of the last bound CpG is updated from *c*_*i*_ to *c*_*i*+*j*_, if (*c*_*i*_+1)+3<*c*_*i*+*j*_.

#### Coefficients of relative enrichment

In [13] we also, as a corollary, introduced the pulldown efficiency, *E*(*n* CpGs), for DNA fragments with *n* accessible mCpGs as the ratio between the fraction of the SssI Control data with *n* CpGs and the fraction of the Input Control data with *n* CpGs. Then *C*_*n*_=*E*(*n*)/*E*(0) represents how much more likely a fragment with *n* mCpGs will be sequenced in the SssI Control data set than a fragment with 0 mCpGs. Hence, as we sum over the fragments that could align to location *x*, this factor accounts for relatively how often we should expect to see a fragment with that many mCpGs. See “Modeled pulldown from SssI-treated DNA” subsection of the Methods for the specific values calculated.

#### Length distribution of DNA fragments

**Fig. 2:**
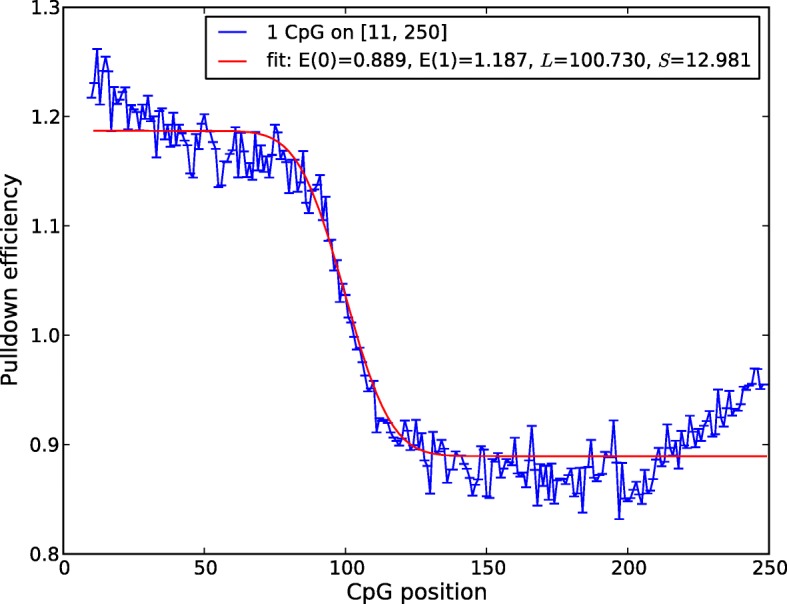
Determining the pulldown fragment length distribution. Pulldown efficiency among read alignments with only 1 CpG within 250 bp downstream as a function of CpG position (with respect to the 5^′^-end). Pulldown efficiency decreases around the CpG position corresponding to the average sequenced fragment length since the CpG represented in the genome is no longer likely to be contained on the read that aligned upstream. Fit to error function (red) is shown on empirical data (blue)

The length distribution of DNA fragments that are aligned to the reference genome can be approximated by Bioanalyzer analysis on the pulldown library after fragmentation. To get a more precise description of the fragment length distribution, we compared the distribution of alignments from the SssI Control to the distribution in the Input Control. Let the fragment length probability distribution be approximated by a Gaussian, *P*(*ℓ*)∼*N*(*L,S*), where *L* is the average fragment length and *S* is the standard deviation. To determine its parameters *L* and *S*, we take all reads that have been aligned to the reference genome and then extend them to a segment of length *ℓ*_max_=250 (larger than the expected actual fragment length) and then consider only those segments where the genomic sequence contains only a single CpG. To avoid edge effects we in addition require that the C of this CpG must be located at, or downstream of, the 11th nucleotide from the 5’ end of the fragment. Let then *p*(1 CpG,*t* nt) be the fraction of segments observed in the SssI Control data set with one CpG, which starts at the *t*th nucleotide (with respect to the 5’-end). Similarly, we define *q*(1 CpG,*t* nt) for the Input Control data set. Then the predicted pulldown efficiency for reads that are sequenced and align to a position with one CpG located at the *t*th nucleotide downstream with respect to that position is:

2 $$\begin{array}{@{}rcl@{}} \frac{p(1 \text{ CpG}, t\ \text{nt})}{q(1\ \text{CpG}, t\ \text{nt})} &=& \sum\limits_{\ell=\ell_{\min}}^{t} R_{0} P(\ell) + \sum\limits_{\ell=t+1}^{\ell_{\max}} R_{1} P(\ell) \\  &=& R_{0} + (R_{1} - R_{0}) P(\ell \ge t+1), \end{array} $$

where *R*_0_ and *R*_1_ represent the pulldown efficiency for fragments with 0 and 1 CpGs, as a ratio of fractions of the pool of reads with only 1 CpG between 11 bp and 250 bp from the 5^′^-end. This expression captures how, as we look at fragments with a single CpG further and further downstream of the 5^′^-end, we will find the length past which the CpG is not likely to actually be on the fragment and not contribute to that fragment’s pulldown probability, and therefore it marks the typical length of the sequenced fragments. For a Gaussian *P*(*ℓ*), we can approximate $P(\ell \ge t) \approx \text { Erf}\left (\frac {t - L}{S \sqrt {2}}\right) - \text { Erf}\left (\frac {1 - L}{S \sqrt {2}}\right)$. We fit the experimental data to Eq. (2) using the Python SciPy function curve_fit to perform least-squares optimization, obtaining parameters (*R*_0_=0.889,*R*_1_=1.187,*L*=100.7,*S*=12.98) from an initial guess of (1.0,1.0,200,50), (Fig. 2). This completely characterizes the length distribution *P*(*ℓ*) and from this we set *ℓ*_min_=3 and *ℓ*_max_=200.

When comparing the fit to the data, we notice an increase over the expected pulldown efficiency at *ℓ*=10∼30. It is not clear what the source of this trend is, though there is another similar increase for *ℓ*>200. We found the latter to be an artifact of our maximum *ℓ* cutoff; when we shifted *ℓ*_max_ from 250 to 300, the increase at the largest *ℓ* shifted with it and the parameter fits for *L* and *S* were not significantly changed. We also note that the values for *R*_0_ and *R*_1_ do not match those for *E*(0 CpGs) and *E*(1 CpG) because the latter are normalized to the larger pool of all fragments with no CpGs contained in the first 10 bases versus the subset with just one CpG within 250 bp downstream of the alignment start.

### Fragment length and mCpG number are sufficient to model pulldown alignment to a genomic window

**Fig. 3:**
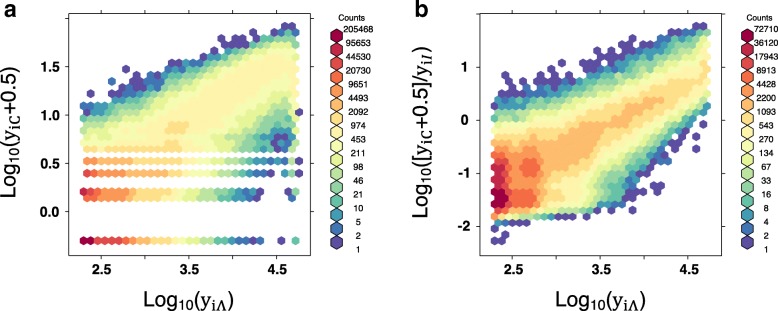
Modeled SssI pulldown coverage, $\sum \nolimits _{x \in i} \Lambda _{x}$, correlates with observed SssI pulldown coverage. Among 100 bp windows on chromosome 7 with at least 75% mappable bases, we calculate expected SssI Control window coverage, $y_{i\Lambda } = \sum \nolimits _{x \in i} \Lambda _{x}$, from our model of pulldown alignments derived from previous MBD pulldown experiments. **a** A density plot shows that *y*_*iΛ*_ correlates with observed SssI pulldown coverage, *y*_*iC*_, represented with offset 0.5 to allow for log-log plotting. **b** To account for parts of the genome that are less likely to be sequenced, we compare *y*_*iΛ*_ to *y*_*iC*_/*y*_*iI*_ – wherever *y*_*iI*_, the read coverage from the Input Control sample, is nonzero – which scales with the pulldown efficiency of genomic window *i*

To begin thinking about using our model of *Λ*_*x*_ as a substitute for experimentally observed MBD pulldown alignments, we wish to see how well SssI Control window coverage correlates with modeled window coverage. We calculated expected SssI Control alignments, *Λ*_*x*_, for every site in chromosome 7 and summed it over 100 bp non-overlapping windows to generate modeled SssI Control window coverage, $y_{i\Lambda }= \left \lfloor \sum \nolimits _{x \in i} \Lambda _{x} \right \rfloor $. Using the same mappability cutoff as in [14], we compare this quantity to the observed SssI Control window coverage, *y*_*iC*_, at every genomic window *i* that has at least 75% mappable bases. In Fig. 3a, there is a general increase in the SssI Control coverage as the modeled SssI Control coverage increases (Pearson correlation between *y*_*iΛ*_ and *y*_*iC*_ is 0.78). Wondering at the reason for the slight turnover in SssI Control coverage for modeled coverage values log10(*y*_*iΛ*_)>4.5, we suspect these regions are more likely to have high GC content and were therefore less likely to be sequenced in these experiments [24]. We can control for which windows are likely to be sequenced by dividing the SssI Control coverage by the Input Control Coverage, *y*_*iI*_, to essentially obtain an unnormalized pulldown efficiency of reads that overlap the window. Comparing that to the modeled SssI Control coverage, we see a positive correlation between a window’s SssI Control coverage and pulldown efficiency at these larger values of *y*_*iΛ*_ (Fig. 3b).

### Model of pulldown alignment improves estimates of methylation

#### Methylation prediction

To predict methylation level from MBD pulldown and assess our model of predicted SssI Control coverage, we use the previously published program BayMeth [14]. BayMeth uses a Bayesian framework to quantify methylation fraction on genomic windows from data from pulldown experiments – done on both a Sample of interest (*S*) that has undergone MBD pulldown but not SssI treatment and an SssI Control (*C*) version of that sample. For each genomic window *i*, the probability of the observed read counts from the Sample of Interest (*y*_*iS*_) and the SssI Control sample (*y*_*iC*_) update the prior distribution for the methylation fraction *μ*. The read counts are assumed to be Poisson-distributed with average read density scaled by parameter *λ*_*i*_, which represents the expected read density for a window of the same CpG density at full methylation. There are two main modes of BayMeth that we compare and modify in this study. The first uses pulldown read coverage from both the Sample of interest and the SssI Control sample (what we call BayMeth-SssI); this is the mode recommended by the authors of BayMeth. The second only uses pulldown data from the Sample of interest (BayMeth-noSssI), which is of use if a matching SssI Control sample is not available. The new implementation that we test here is to run BayMeth with expected read coverage, $y_{i\Lambda } = \left \lfloor \sum \nolimits _{x \in i} \Lambda _{x} \right \rfloor $, calculated by our model developed above, as a proxy for the SssI-treated Control read coverage *y*_*iC*_; we call this mode BayMeth-calcSssI, which can be used even in the absence of a real SssI-treated Control sample. While this formulation of *y*_*iΛ*_ only explicitly models reads that start or end in window *i* – in contrast to the window coverage described by *y*_*iC*_ – we will find that including modeled reads spanning, but not starting in, a window does not meaningfully improve predictions by BayMeth-calcSssI, even when the window width is much smaller than the average fragment length.

#### Methylation predictions on 100 bp windows

The methylation predictions generated by BayMeth-SssI, BayMeth-noSssI, and BayMeth-calcSssI are each assessed against the methylation predictions measured by RRBS, $\mu _{i}^{\text {RRBS}}$, for a Sample of interest from [22]. We set a window’s methylation state to be methylated if its RRBS methylation is >0.50 and unmethylated if it is ≤0.50. Then ROC curves are generated and we ultimately evaluate each method’s performance in separating methylated from unmethylated windows through its corresponding AUC.

**Fig. 4:**
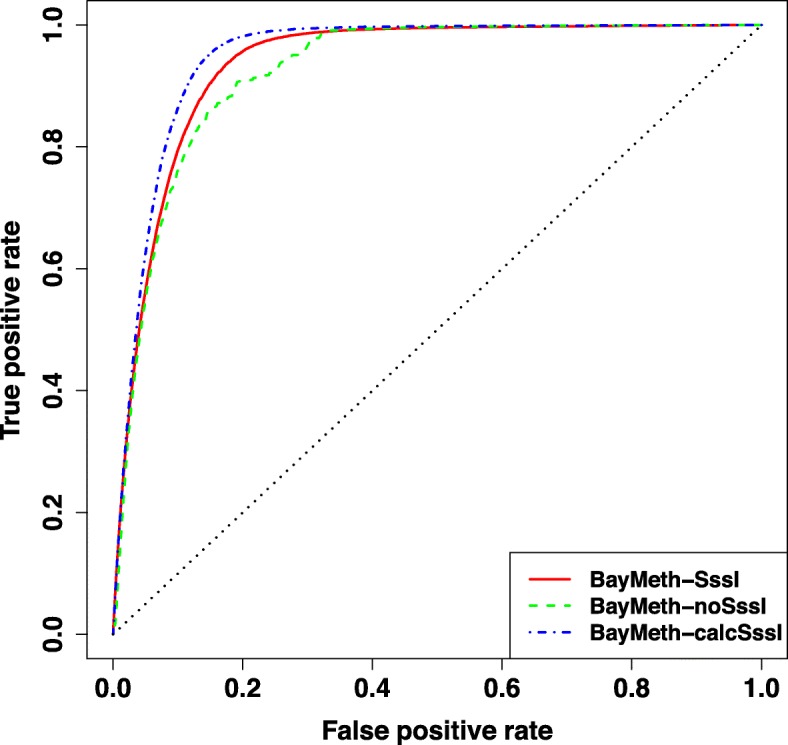
Assessing methylation predictions genome-wide on 100 bp windows. For all 100 bp windows on hg18 with at least 75% mappable bases and with at least 10 reads of RRBS coverage, the ROC curve is plotted to assess the methylation predictions from three configurations of BayMeth. The *y*=*x* line is marked in dotted black. The larger the area under the curve (AUC), the better the method serves as an indicator of a window’s methylated/unmethylated state: BayMeth-calcSssI (0.948) > BayMeth-SssI (0.936) > BayMeth-noSssI (0.925)

We first compare the performance of BayMeth-calcSssI on all 100 bp windows on hg18 that pass the minimum RRBS coverage and mappable base percentage (212,252 windows out of 14,506,245 total windows with at least one annotated CpG). In Fig. 4, the ROC curves for BayMeth-SssI, BayMeth-noSssI, and BayMeth-calcSssI are plotted, showing that BayMeth-calcSssI has the largest AUC (0.948) followed by BayMeth-SssI (0.936) and BayMeth-noSssI (0.925).

For each method’s most efficient ordering (the cutoff corresponding to the point on the ROC curve furthest from the *y*=*x* line), 94.5*%* of methylated windows are correctly categorized by BayMeth-calcSssI, a ∼ 5*%* improvement over BayMeth-noSssI, and ∼ 1*%* improvement over BayMeth-SssI. On the reverse, 14.3*%* of unmethylated windows are incorrectly categorized by BayMeth-calcSssI, a ∼ 5*%* improvement over BayMeth-noSssI, and ∼ 3*%* improvement over BayMeth-SssI.

**Fig. 5:**
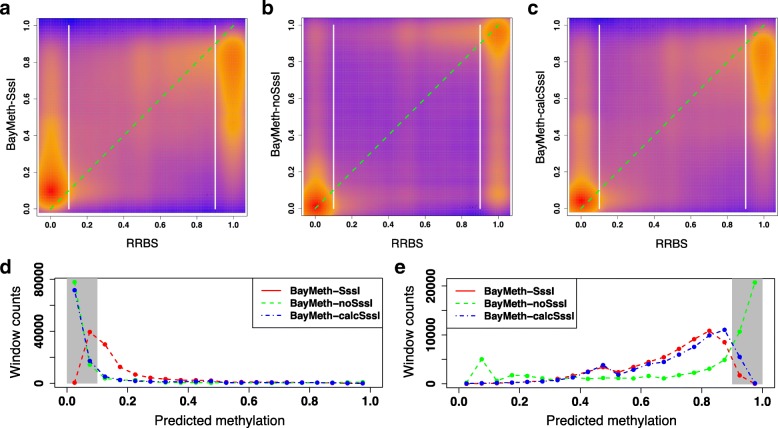
Profile of methylation predictions on 100 bp windows. For the same subset of windows as analyzed in Fig. 4, smoothed density plots compare the window methylation as calculated by RRBS to the methylation predicted by (**a**) BayMeth-SssI, (**b**) BayMeth-noSssI, and (**c**) BayMeth-calcSssI. The *y*=*x* line is plotted in dashed green and divisions at RRBS =0.1 and RRBS =0.90 are plotted in bolded white. The distribution of methylation predictions given by each configuration is plotted for windows with an RRBS methylation (**d**) ≤0.10 and (**e**) ≥0.90, the methylation regimes (blocked in gray) where most windows lie

To get a sense of what methylation states each method is better at predicting, smoothed density plots in Fig. 5 compare predicted methylation levels to their RRBS methylation. First, methylation level among 100 bp windows with RRBS coverage is highly bimodal, similar to the mean methylation levels observed at individual CpGs. There are 55% more unmethylated windows than methylated windows, but as both methylated and unmethylated states are well-represented in this RRBS sample, an indicator of methylation state has to achieve high accuracy in both regimes. About 81% of plotted windows have an RRBS methylation level that is ≤0.10 or ≥0.90. From Fig. 5a-c, we see that BayMeth-SssI and BayMeth-calcSssI give less precise predictions to windows with medium levels of methylation than those given by BayMeth-noSssI. For windows with RRBS methylation level ≤0.10 (Fig. 5d), more windows are predicted by BayMeth-noSssI to still have a methylation level ≤0.10 than by BayMeth-SssI, and BayMeth-noSssI also miscategorizes fewer windows overall (4.80*%* versus 5.50*%*, of windows with $\mu _{i}^{\text {RRBS}} \leq 0.10$). Among windows with an RRBS methylation level of at least 0.90 (Fig. 5e), more windows are predicted by BayMeth-noSssI than by BayMeth-SssI to have a methylation level ≥0.90. However, overall BayMeth-noSssI ends up miscategorizing more of these high-methylation windows than BayMeth-SssI because of how many more windows it predicts with a methylation level ≤0.50 (23.3*%* versus 15.9*%*, of windows with $\mu _{i}^{\text {RRBS}} \geq 0.90$). Interestingly, the methylation prediction profile of BayMeth-calcSssI is most similar to BayMeth-SssI in the high-methylation regime (and miscategorizes the lowest percentage of these windows at 15.5*%*) and most similar to BayMeth-noSssI in the low-methylation regime (and again miscategorizes the lowest percentage at 4.07*%*). Thus, BayMeth-calcSssI appears to capture the strengths of the other two BayMeth configurations and, on this scale, perform better than both of them.

#### Methylation predictions on 10 bp windows

**Fig. 6:**
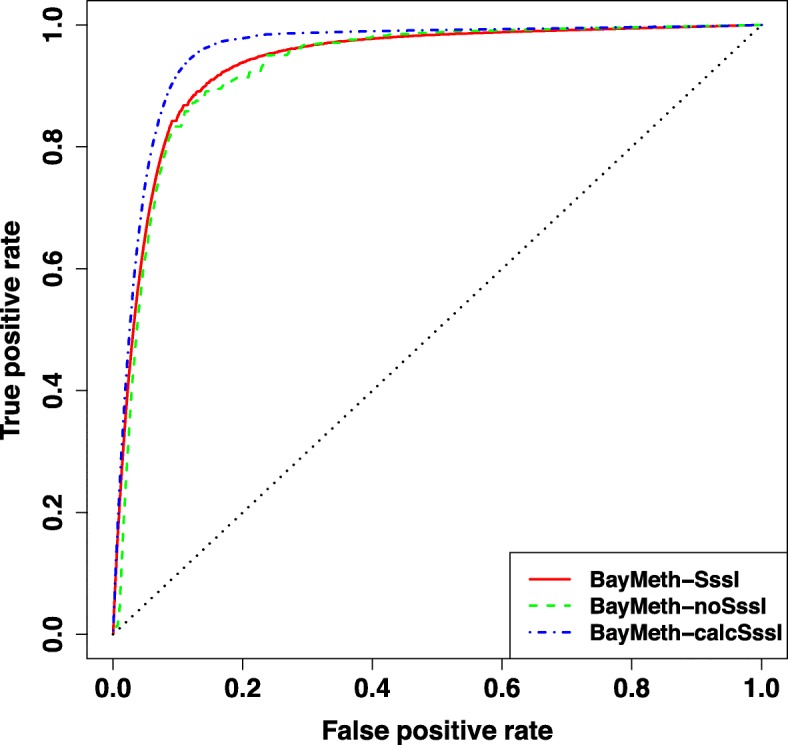
Assessing methylation predictions genome-wide on 10 bp windows. For all 10 bp windows on hg18 with at least 75% mappable bases and with at least 10 reads of RRBS coverage, the ROC curve is plotted to assess the methylation predictions from three configurations of BayMeth. The *y*=*x* line is marked in dotted black. These AUCs are as least as large as those for 100 bp windows, and again BayMeth-calcSssI produces the largest AUC: BayMeth-calcSssI (0.955) > BayMeth-SssI (0.936) > BayMeth-noSssI (0.930)

Since one of the advantages of RRBS is single CpG resolution in methylation predictions, we explore the accuracy of methylation predictions informed by MBD pulldown on 10 bp windows. Of the 25,858,448 windows of width 10 bp on hg18 with at least one annotated CpG, 457,335 pass the minimum mappable base percentage (75%) and RRBS coverage (10). Of these windows, 63% contain only one CpG, 29% contain two CpGs, and the remaining 8% have three or more. Evaluating the three methylation prediction methods on these windows, the ROC curves for BayMeth-SssI, BayMeth-noSssI, and BayMeth-calcSssI are plotted in Fig. 6. Again, the AUC for BayMeth-calcSssI (0.955) is highest followed by BayMeth-SssI (0.936) and BayMeth-noSssI (0.930). These AUCs are even higher than they were for the 100 bp windows in the case of BayMeth-calcSssI and BayMeth-noSssI.

**Fig. 7:**
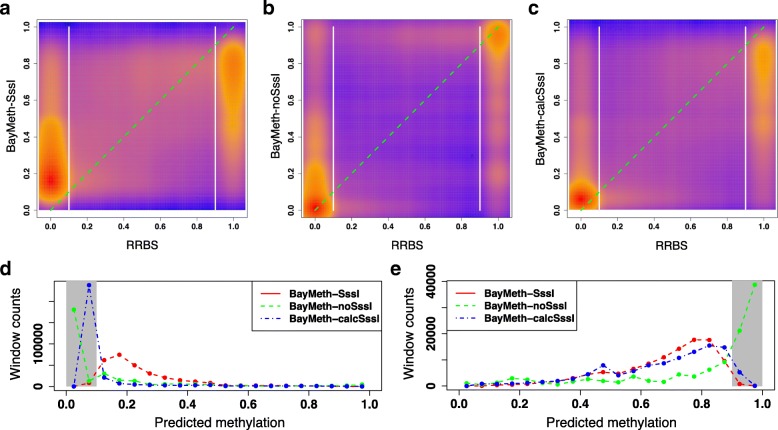
Profile of methylation predictions on 10 bp windows. For the same subset of windows as analyzed in Fig. 6, smoothed density plots compare the window methylation as calculated by RRBS to the methylation predicted by (**a**) BayMeth-SssI, (**b**) BayMeth-noSssI, and (**c**) BayMeth-calcSssI. The *y*=*x* line is plotted in dashed green and divisions at RRBS =0.1 and RRBS =0.90 are plotted in bolded white. The distribution of methylation predictions given by each configuration is plotted for windows with an RRBS methylation (**d**) ≤0.10 and (**e**) ≥0.90, the methylation regimes (blocked in gray) where most windows lie

Smoothed density plots of the predicted methylations versus the RRBS methylation are shown in Fig. 7a-c. The percentage of windows with extremal RRBS methylation levels (≤0.10 or ≥0.90) increases to 88%. As with the methylation predictions on 100 bp windows, BayMeth-calcSssI and BayMeth-noSssI provide similar distributions of methylation predictions for windows in the low methylation regime, while BayMeth-calcSssI and BayMeth-SssI provide similar distributions of predictions for windows in the high methylation regime (Fig. 7d-e). Though, on this window size, BayMeth-calcSssI miscategorizes the most windows in the high methylation regime (20.6*%*), followed by BayMeth-SssI (16.1*%*), and BayMeth-noSssI (15.9*%*). However, in the low methylation regime, BayMeth-calcSssI performs best, categorizing 96.9*%* of windows correctly, followed by BayMeth-SssI (95.1*%*), and then BayMeth-noSssI (94.3*%*).

### Testing parameter robustness on chromosome 7

With these results, we must also make sure that the quality of BayMeth-calcSssI predictions is robust to variation of the input parameters involved in calculating the modeled SssI Control coverage, *y*_*iΛ*_. We use chromosome 7 to test the sensitivity of the AUC measure since it was the chromosome reported on in [14]. On chromosome 7, 11,158 windows of width 100 bp meet the minimum mappable base percentage and RRBS coverage (which represents 1.4*%* of windows on chromosome 7 with at least one CpG). Running our three configurations of BayMeth with the previously stated parameters, we find that methylation predictions from the BayMeth-calcSssI method produced the largest AUC (0.946), followed by BayMeth-SssI (0.938) and BayMeth-noSssI (0.925). If we, instead, reduce the minimum separation between consecutive CpGs to 2 bp (from 3 bp) to potentially count additional CpGs as “accessible” (0.946); scale the overall depth normalization, i.e. the prefactor in Eq. (1), by 10 (0.946), or by 0.10 (0.946); or collapse the fragment length distribution to the determined average fragment length (0.945), i.e. *P*(*ℓ*)∼*δ*(*ℓ*−*ℓ*_avg_); the AUCs calculated after each of these individual modifications all remain within 0.001 of the initial model. Additionally, we considered whether information is effectively lost by only summing the *Λ*_*x*_ terms for sites, *x*, in window *i*, and thus only modeling contributions from reads that start in window *i* (on either the forward or reverse strand). To instead model a sum over all reads that likely overlap window *i*, we tested using a fixed fragment length (*P*(*ℓ*)∼*δ*(*ℓ*−*ℓ*_avg_)) and, for the proxy SssI Control measure, summing over all sites and strands where a fragment of length *ℓ*_avg_ would overlap window *i*:

3 $$ {y_{i\Lambda} = \left \lfloor \left(\sum\limits_{\underset{\text{overlapping window}\ i}{[x, x + (\ell_{\text{avg}} - 1)]}} \Lambda_{x,+}\right) + \left(\sum\limits_{\underset{\text{overlapping window}\ i}{[x-(\ell_{\text{avg}} - 1), x]}} \Lambda_{x,-} \right)\right \rfloor,}  $$

where we use *Λ*_*x*,+_ to refer to the expected pulldown to position *x* from the forward strand, and similarly for *Λ*_*x*,−_ for the reverse strand. This formulation also produces an AUC of 0.945. Additionally, this formulation and each of the other modifications to the BayMeth-calcSssI method applied to analysis of chromosome 7 on 10 bp windows also produced methylation prediction profiles that all had an AUC within 0.001 of the AUC produced by the original method.

### Model of pulldown alignment can be applied to methylation predictions on external experiments done with the same MBD protein

**Fig. 8:**
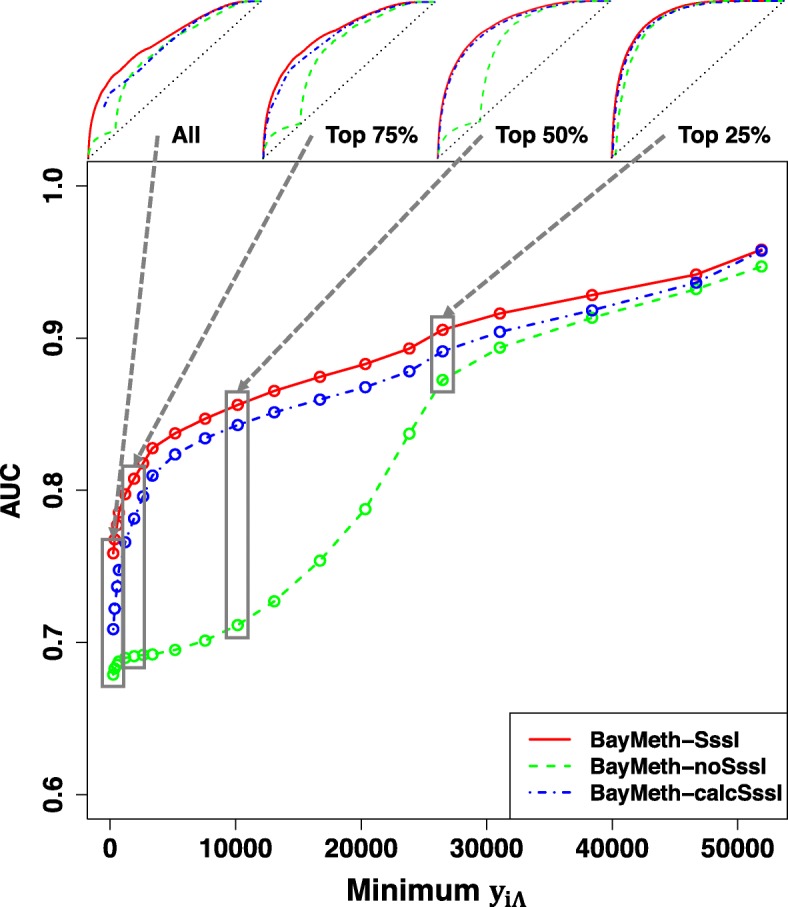
Methylation predictions for an external MBD/WGBS data set. To act as an external data set to test the effectiveness of our MBD pulldown model, we use the WGBS and MBD pulldown data from [14]. We consider all 100 bp windows on chromosome 7 with at least 75% mappable bases and at least 33 reads in WGBS coverage, the same specifications as considered by Riebler et al.. Each plot point in the main graph corresponds to a 5 percentile increment in the minimum modeled SssI Control coverage, *y*_*iΛ*_, from the 0^th^ percentile to the 95^th^ percentile, and the AUC is calculated for each BayMeth configuration on all windows that meet the minimum threshold. ROC curves analyzing the windows with the top quartiles in *y*_*iΛ*_ are plotted above, corresponding to the cutoff identified in the gray boxes on the main graph

For our model of expected pulldown alignments, *Λ*_*x*_, to be of general use in predicting methylation level from MBD pulldown experiments, we must show that it may be applied to pulldown experiments that it has not been trained on. To that end, we adapted our model to be used in place of the SssI Control data from the human fibroblast (IMR-90) sample analyzed in Riebler et al. [14]. We used the average fragment length of 300 bp given in that study to set the fragment length probability distribution to *P*(*ℓ*)=*δ*(*ℓ*−300 bp), since the width of that distribution was not described, and kept all other parameters the same. On chromosome 7, 426,366 windows of width 100 bp pass both the 75% minimum mappable base percentage and the minimum WGBS coverage, set in that study to 33. Applying the three configurations of methylation prediction on these windows, the ROC curves for BayMeth-SssI, BayMeth-calcSssI, and BayMeth-noSssI are plotted in the top leftmost panel of Fig. 8 (“All” windows). The BayMeth-SssI mode performs best with an AUC of 0.763, followed by BayMeth-calcSssI (0.715) and BayMeth-noSssI (0.681). Since the AUC produced by BayMeth-calcSssI on this set of windows is not as high as we have seen in the previous sections, it would be convenient to have a quantity that indicates for each window whether BayMeth-calcSssI is likely to provide a good methylation prediction. A suitable choice is the calculated SssI Control window coverage, *y*_*iΛ*_, itself. We had developed our model for SssI Control pulldown alignments by considering what DNA fragment features significantly change the pulldown efficiency, and the calculated SssI Control coverage approximates how efficiently, relatively, an MBD pulldown experiment should be expected to sample reads from that window. In the Bayesian framework used in BayMeth, the windows that it samples the most information from should be predicted on more accurately. To test the usefulness of calculated SssI Control coverage as an indicator of BayMeth-calcSssI performance, we range through different minimum cutoffs on *y*_*iΛ*_ (in steps of 5 percentile) and calculate ROC curves on all windows above that threshold. We see in the main body of Fig. 8 that the AUCs for the three methods increase monotonically with the minimum window *y*_*iΛ*_. For cutoffs above the 85th percentile in SssI Control coverage, the AUCs from all three methods are above 0.90.

Considering this framework for assessment, then, we see that the quality of methylation predictions produced by BayMeth-SssI and BayMeth-calcSssI follow each other closely and their AUC values stay within 0.05 of each other at all cutoffs. The AUCs for BayMeth-noSssI get within 0.05 only for a minimum modeled SssI Control coverage ≥75^th^ percentile, and at all cutoffs, BayMeth-calcSssI performs better than BayMeth-noSssI. Thus, in the absence of SssI Control data, our model improves methylation predictions. While additional comparisons to external data sets would be beneficial in showing our model’s broad applicability, MBD-seq data sets are not often generated with an SssI Control, which happens to be something our method attempts to rectify. More pressingly, genome-wide bisulfite sequencing data (either RRBS or WGBS) that can act as a truth data set against an MBD-seq data set is not usually available simultaneously for the same samples. Thus, to our knowledge there are no other MBD-seq/BS samples that use the same protein domain for enrichment, are of sufficient sequencing quality, and whose results would not be potentially confounded by copy number variation.

## Discussion

We developed a model for the number of reads aligned to genomic location *x* from an MBD pulldown experiment done on SssI-treated DNA. The model is based on the expected number of mCpGs to be captured on fragments taken from that location and the relative representation of reads in a pulldown library given the number of accessible mCpGs. Summed along all positions in a genomic window, it correlates with observed SssI data. MBD pulldown coverage from a fully-methylated sample can be used to calibrate the expectation of MBD pulldown read density from a Sample of interest. Thus, we tested our model insofar as it improves statistical methods that convert MBD pulldown coverage on a genomic window to an estimate of absolute methylation level. The algorithm BayMeth is one approach for obtaining methylation estimates from MBD pulldown data that improves upon previous algorithms, as well as the MEDIPS algorithm that incorporates methylated DNA immunoprecipitation sequencing (MeDIP-seq) data instead of protein pulldown data. BayMeth fits parameters to the distribution of mean read coverage at full methylation by sampling within each CpG density class, and has two main configurations that we call BayMeth-SssI (simultaneously models pulldown from the Sample of interest and the SssI Control, to be used if such a control sample is available) and BayMeth-noSssI (only models pulldown from the Sample of interest and thus applicable if no control sample is available). Here, we added the configuration BayMeth-calcSssI by calculating SssI Control coverage for each genomic window, *y*_*iΛ*_, using our model and substituting it for the observed SssI Control input, *y*_*iC*_ (applicable in the absence, but still providing the benefits, of a control sample).

Comparing methylation predictions for a sample of interest from BayMeth-SssI, BayMeth-noSssI, and BayMeth-calcSssI against RRBS estimates for the same sample, we find that the profile of estimates from BayMeth-calcSssI produces the largest AUC for both 100 bp and 10 bp windows. One possible interpretation of this finding is that our model is overfitting the data. There are two reasons why this is highly unlikely. First, and most importantly, fitting the model to measured SssI data is completely independent of the AUC calculations used to evaluate the performance of the model in the context of the BayMeth framework. Thus, the model fit *cannot* be influenced by optimization of the AUC, thereby not even allowing the possibility of overfitting. Second, in the case of overfitting, small changes to the model should remove the overfitting advantage and lead to a clear decrease in performance. On the contrary, our robustness studies on chromosome 7 indicate that the AUC is very stable under various modeling choices. That leaves the question of how else one could explain that the model performs better than the actual experimental SssI data. Performing better than the BayMeth-noSssI indicates that the modeled SssI Control data in BayMeth-calcSssI adds more information than what can be inferred from the distribution of pulldown coverage, *y*_*iS*_, in each CpG density class. While our model of MBD pulldown is necessarily a simplification of the true SssI Control experiment, it does model an SssI Control experiment with very high depth of coverage, one on the order of 6×10^10^ reads. As a result, windows with high modeled SssI Control coverage, *y*_*iΛ*_, with low coverage from the Sample of interest, *y*_*iS*_, are more easily predicted to have lower methylations, with lower variance, in the BayMeth-calcSssI configuration. The increased scale also allows for finer distinction between windows of similar CpG densities. In summary, in contrast to the experiment, the modeled SssI control does *not* suffer from sampling uncertainties, which might explain how BayMeth-calcSssI is able to outperform BayMeth-SssI.

At the same time, compared on our RRBS data set, each method produces an AUC greater than 0.90, suggesting each can act as a reliable indicator of methylated/unmethylated state. The differences among the three methylation prediction configurations are more pronounced in the external MBD pulldown and WGBS data sets from the IMR-90 sample analyzed in [14]. Over all the 100 bp windows on chromosome 7 meeting the minimum WGBS coverage and mappable base percentage, no configuration produces an AUC greater than 0.80. Considering the weaker performance of BayMeth-calcSssI on this data set relative to BayMeth-SssI, there is the question of whether the parameters calibrated from our earlier experiments do not carry over to this one. First, we ask if BayMeth-calcSssI applied to the external data set at least does well when compared on a set of windows representative of an RRBS data set. To that end, if we look at the windows that were analyzed in both the WGBS and RRBS data sets (8998 windows on chromosome 7 that also matched the minimum mappability), the AUCs achieved by all three methods are greater than 0.95 with BayMeth-SssI (0.975) being the highest, though it differs from BayMeth-calcSssI (0.973) less than BayMeth-calcSssI does from BayMeth-noSssI (0.957). The improvement should be expected because RRBS biases the resulting data set toward regions of higher CpG density, whereas WGBS attempts to represent all parts of the genome, and at least those parts with high mappability. Of course, a window with higher CpG density would correlate with a higher calculated SssI Control coverage, *y*_*iΛ*_. As a result, windows in the RRBS data set are on the highest end of modeled SssI Control coverage: The 10^th^ percentile value of *y*_*iΛ*_, among mappable windows with RRBS coverage, is the 93^rd^ percentile value among all mappable windows with at least 1 CpG. Second, we ask how predictions by BayMeth-calcSssI relate to those by BayMeth-SssI over the range of cutoffs in *y*_*iΛ*_. Given that in Fig. 8, the strength of predictions produced by BayMeth-calcSssI tracks those by BayMeth-SssI very closely, it further suggests that the *Λ*_*x*_ model has not drastically broken down when used for this experiment. More likely, the inclusion of lower CpG density windows (and hence, generally lower *y*_*iΛ*_ values) in the analysis of this external data set, is what weakens each method as an indicator of methylated/unmethylated state as to be expected from the relatively low gain in pulldown from a single isolated CpG (compare *C*_1_∼1.49 to *C*_3_∼32). Thus, this is likely a shortcoming of the MBD pulldown method in general in quantifying low CpG density regions. In applying our method to data sets enriched for low-CpG dense regions, an easy choice of *y*_*iΛ*_ for a cutoff may not be clear at the outset. We recommend using a 2D-density plot of the variance on the posterior probability calculation (an automatic output of BayMeth) versus *y*_*iΛ*_ to identify a threshold in *y*_*iΛ*_ above which the variance is relatively flat (Additional file 2: Figure S1). Estimating a cutoff in this way for the WGBS data places the *y*_*iΛ*_ threshold between the top 25% and 30% of values, with AUCs >0.85 on the corresponding data (Additional file 2: Figure S1).

How generally applicable our model and its parameters are is an important concern. Of course, in an ideal world, every pulldown experiment would be paired with an SssI experiment performed under the exact same conditions. But in reality this is not always feasible, as reflected in the scarcity of publicly available SssI data. We will thus discuss here the transferability of our model between different experiments. Our model is characterized by three ingredients, the pulldown coefficients *C*_*n*_, a scheme to determine accessible CpGs from the positions of genomically encoded CpGs, and the mean and standard deviation of the fragment length distribution. Given that most modern high throughput sequencing protocols use paired-end protocols, the fragment length distribution is directly obtainable from each library. For single-end libraries, retaining the bioanalyzer traces typically obtained during quality control of library preparation would provide the same information at somewhat lower resolution [22]. Thus, the fragment length distribution can be easily adapted to a new sequencing experiment, as we have done here in our analysis of the Riebler et al. data. The scheme to determine accessible CpGs from the positions of genomically encoded CpGs reflects the minimum distance between two CpGs that can be bound simultaneously by two proteins without steric clashes [13]. It thus represents a fundamental biophysical property of the protein used for the pulldown. It will have to be determined from scratch if a different protein is used for the pulldown, but should otherwise be independent of experimental conditions. In our robustness analysis, we also found that the change from 3 bp minimal distance to 2 bp minimal distance did not affect performance measurably, indicating that at least small changes in the bulkiness of the protein are tolerable. The pulldown coefficients *C*_*n*_ are the most sensitive ingredients of the model. They could vary with experimental conditions and certainly will vary with the protein used for the pulldown. The fact that we could successfully apply our set of coefficients to the data sets of Riebler et al. (at least on the high CpG density regions, where pulldown sequencing is more successful as a whole as discussed in the previous paragraph) shows that it is possible to transfer the model between data sets generated under different experimental conditions as long as the same protein is used. In addition, as our model captures the fundamental interactions between the protein and the methylated DNA, we do not expect the model to be sensitive to sequence features other than the locations of the CpGs. We thus expect that our model would allow one to generate calculated SssI data of similar quality for any organism based on its genome alone, even if no experimental SssI data sets for this organism were available.

## Conclusion

We built a model of MBD pulldown alignments from SssI-treated DNA using empirically-derived relationships from our previous studies on MBD pulldown experiments [13]. Quantifying absolute methylation level is an important step in interpreting results from MBD pulldown experiments and in allowing results to be compared across different experiments. We used our pulldown alignment model to calculate pulldown coverage of SssI-treated DNA and substituted it for the use of observed SssI Control pulldown in the implementation of the program BayMeth [14]. As determined by its authors, BayMeth performs best when it is run with SssI Control data. Against RRBS-determined methylation levels calculated genome-wide, BayMeth informed by our SssI pulldown model showed improvements as an indicator of methylated/unmethylated state, over BayMeth informed by observed SssI pulldown. This held even when we looked at methylation predictions on 10 bp windows, a scale at which a majority of windows only contain a single CpG. Looking specifically at how well each configuration of BayMeth did at classifying genomic windows with extremal methylation levels, BayMeth informed by our model seemed to combine the particular capacities of the other two BayMeth configurations: that of BayMeth with observed SssI data on classifying windows with high methylation levels, and that of BayMeth with no SssI data on classifying windows with low methylation levels. To see if our model parameters and performance could extend to external data sets and therefore be of general use, we generated methylation predictions on a Sample of interest from [14], only updating the average fragment length parameter to match the MBD pulldown data from this sample. Against methylation estimates calculated from WGBS data on this sample, all three methods performed worse – as expected on a data set with more low CpG density windows. On this data set, BayMeth with SssI data performed best among the three configurations, but BayMeth with our modeled SssI data always did better than BayMeth run without any SssI estimate. Furthermore, we found that the SssI Control pulldown coverage calculated by the model was itself a good indicator of whether BayMeth supplemented by our model would infer a good estimate on that window. Thus, in the absense of an SssI Control pulldown data set, our modeled data is likely to improve methylation predictions, potentially even over predictions made with real SssI data, especially on windows with higher CpG density.

## Additional files

Additional file 1Standard deviation calculation on MBD pulldown scale factors. **Table S1** giving the details of the per-chromosome sub-sampling process to calculate a sample-to-sample standard deviation on the MBD pulldown scale factors listed in Table 1. The per-chromosome read alignment counts, per number of well-separated CpGs, in each data set (Pulldown and Background Control) are shown at top, with the resulting calculation of *C*_*n*_ values on each chromosome, the chromosome-averaged $C_{n}^{*}$ values, the per-chromosome calculation of fluctuations around this average ($(C_{n} - C_{n}^{*})^{2}$), and finally the standard deviation *s*_*ss*_ on each *C*_*n*_. (XLSX 26 kb)

Additional file 2 Selecting a cutoff in *y*_*iΛ*_. **Figure S1**. Among windows in the Riebler et al. pulldown data set passing mappability and WGBS thresholds, we plot (a) the variance on the posterior probability of methylation from BayMeth-SssI against the measured SssI, which shows a similar decay profile but a difference in distribution from the plot of the (b) variance from BayMeth-calcSssI against *y*_*iΛ*_, our calculated SssI. In isolating a subset on which the methylation predictions can be more trusted when running BayMeth-calcSssI, we select a cutoff in *y*_*iΛ*_ (dashed line) based on a region of stable variance on the prediction itself. The (c) ROC curves resulting from the methods being run on this subset with the AUC printed in the corresponding color. (PDF 245 kb)

## Abbreviations

AUC: Area under the (ROC) curve
CpG: Cytosine followed by guanine
DNA: Deoxyribonucleic acid
ENCODE: Encyclopedia of DNA elements
MBD: Methyl-CpG binding domain
MeDIP: Methylated DNA immunoprecipitation
ROC: Receiver operator characteristic
RRBS: Reduced representation bisulfite sequencing
WGBS: Whole genome bisulfite sequencing

## References

[1] Kurdyukov Sergey, Bullock Martyn (2016). DNA Methylation Analysis: Choosing the Right Method. Biology.

[2] Soozangar N, Sadeghi MR, Jeddi F, Somi MH, Shirmohamadi M, Samadi N (2017). Comparison of genome-wide analysis techniques to dna methylation analysis in human cancer. J Cell Physiol.

[3] De Meyer T, Mampaey E, Vlemmix M, Denil S, Trooskens G, Renard J. -P., De Keulenaer S, Dehan P, Menschaert G, Van Criekinge W (2013). Quality evaluation of methyl binding domain based kits for enrichment DNA-methylation sequencing. PLoS ONE.

[4] Weber M, Davies JJ, Wittig D, Oakeley EJ, Haase M, Lam WL, Schubeler D (2005). Chromosome-wide and promoter-specific analyses identify sites of differential DNA methylation in normal and transformed human cells. Nat Genet.

[5] Aberg KA, Chan RF, Xie L, Shabalin AA, van den Oord EJCG (2018). Methyl-CpG-binding domain sequencing: Mbd-seq. Methods Mol Biol.

[6] Hirst M, Marra MA (2010). Next generation sequencing based approaches to epigenomics. Brief Funct Genom.

[7] Verlaat W, Snijders PJF, Novianti PW, Wilting SM, De Strooper LMA, Trooskens G, Vandersmissen J, Van Criekinge W, Wisman GBA, Meijer CJLM, Heideman DAM, Steenbergen RDM (2017). Genome-wide DNA Methylation Profiling Reveals Methylation Markers Associated with 3q Gain for Detection of Cervical Precancer and Cancer. Clin Cancer Res.

[8] Decock A, Ongenaert M, Cannoodt R, Verniers K, De Wilde B, Laureys G, Van Roy N, Berbegall AP, Bienertova-Vasku J, Bown N, Clement N, Combaret V, Haber M, Hoyoux C, Murray J, Noguera R, Pierron G, Schleiermacher G, Schulte JH, Stallings RL, Tweddle DA, De Preter K, Speleman F, Vandesompele J (2016). Methyl-CpG-binding domain sequencing reveals a prognostic methylation signature in neuroblastoma. Oncotarget.

[9] Subhash S, Andersson PO, Kosalai ST, Kanduri C, Kanduri M (2016). Global DNA methylation profiling reveals new insights into epigenetically deregulated protein coding and long noncoding RNAs in CLL. Clin Epigenetics.

[10] Place TL, Fitzgerald MP, Venkataraman S, Vorrink SU, Case AJ, Teoh MLT, Domann FE (2011). Aberrant promoter CpG methylation is a mechanism for impaired PHD3 expression in a diverse set of malignant cells. PLoS ONE.

[11] Aberg KA, Xie L, Chan RF, Zhao M, Pandey AK, Kumar G, Clark SL, van den Oord EJ (2015). Evaluation of Methyl-Binding Domain based enrichment approaches revisited. PLoS ONE.

[12] Nair SS, Coolen MW, Stirzaker C, Song JZ, Statham AL, Strbenac D, Robinson MD, Clark SJ (2011). Comparison of methyl-DNA immunoprecipitation (MeDIP) and methyl-CpG binding domain (MBD) protein capture for genome-wide DNA methylation analysis reveal CpG sequence coverage bias. Epigenetics.

[13] Moreland B, Oman K, Curfman J, Yan P, Bundschuh R (2016). Methyl-CpG/MBD2 interaction requires minimum separation and exhibits minimal sequence specificity. Biophys J.

[14] Riebler A, Menigatti M, Song JZ, Statham AL, Stirzaker C, Mahmud N, Mein CA, Clark SJ, Robinson MD (2014). BayMeth: improved DNA methylation quantification for affinity capture sequencing data using a flexible Bayesian approach. Genome Biol.

[15] Liu Y, Wilson D, Leach RJ, Chen Y (2016). MBDDiff: an R package designed specifically for processing MBDcap-seq datasets. BMC Genomics.

[16] Lan X, Adams C, Landers M, Dudas M, Krissinger D, Marnellos G, Bonneville R, Xu M, Wang J, Huang TH, Meredith G, Jin VX (2011). High resolution detection and analysis of CpG dinucleotides methylation using MBD-Seq technology. PLoS ONE.

[17] Lienhard M, Grimm C, Morkel M, Herwig R, Chavez L (2014). MEDIPS: genome-wide differential coverage analysis of sequencing data derived from DNA enrichment experiments. Bioinformatics.

[18] Down TA, Rakyan VK, Turner DJ, Flicek P, Li H, Kulesha E, Graf S, Johnson N, Herrero J, Tomazou EM, Thorne NP, Backdahl L, Herberth M, Howe KL, Jackson DK, Miretti MM, Marioni JC, Birney E, Hubbard TJ, Durbin R, Tavare S, Beck S (2008). A Bayesian deconvolution strategy for immunoprecipitation-based DNA methylome analysis. Nat Biotechnol.

[19] Lienhard M, Grasse S, Rolff J, Frese S, Schirmer U, Becker M, Borno S, Timmermann B, Chavez L, Sultmann H, Leschber G, Fichtner I, Schweiger MR, Herwig R (2017). QSEA-modelling of genome-wide DNA methylation from sequencing enrichment experiments. Nucleic Acids Res.

[20] Ding J, Bar-Joseph Z (2017). MethRaFo: MeDIP-seq methylation estimate using a Random Forest Regressor. Bioinformatics.

[21] Renbaum P, Abrahamove D, Fainsod A, Wilson GG, Rottem S, Razin A (1990). Cloning, characterization, and expression in Escherichia coli of the gene coding for the CpG DNA methylase from Spiroplasma sp. strain MQ1(M,SssI). Nucleic Acids Res.

[22] Frankhouser DE, Murphy M, Blachly JS, Park J, Zoller MW, Ganbat J. -O., Curfman J, Byrd JC, Lin S, Marcucci G, Yan P, Bundschuh R (2014). PrEMeR-CG: inferring nucleotide level DNA methylation values from MethylCap-seq data. Bioinformatics.

[23] Derrien T, Estelle J, Marco Sola S, Knowles DG, Raineri E, Guigo R, Ribeca P (2012). Fast computation and applications of genome mappability. PLoS ONE.

[24] Oman K. Nucleic acid high-throughput sequencing studies present unique challenges in analysis and interpretation. PhD thesis, The Ohio State University, Department of Physics. 2015.

[25] Moreland B, Oman K, Curfman J, Yan P, Bundschuh R. Data from ‘Detailed characterization of MBD2 pulldown of methylated DNA sequences’. SRA. 2016. https://www.ncbi.nlm.nih.gov/bioproject/PRJNA350318/.

[26] Riebler A, Menigatti M, Song JZ, Statham AL, Stirzaker C, Mahmud N, Mein CA, Clark SJ, Robinson MD. Data from ‘BayMeth: improved DNA methylation quantification for affinity capture sequencing data using a flexible Bayesian approach’. 2014. http://imlspenticton.uzh.ch/robinson_lab/BayMeth/. 10.1186/gb-2014-15-2-r35 PMC4053803 24517713

