# Supplementary figures and images for: A model of pulldown alignments from SssI-treated DNA improves DNA methylation prediction
Source: BMC Bioinformatics. 2019 Aug 19;20:431. doi: 10.1186/s12859-019-3011-2 (PMC6700779; doi:10.1186/s12859-019-3011-2)

# Figure S1

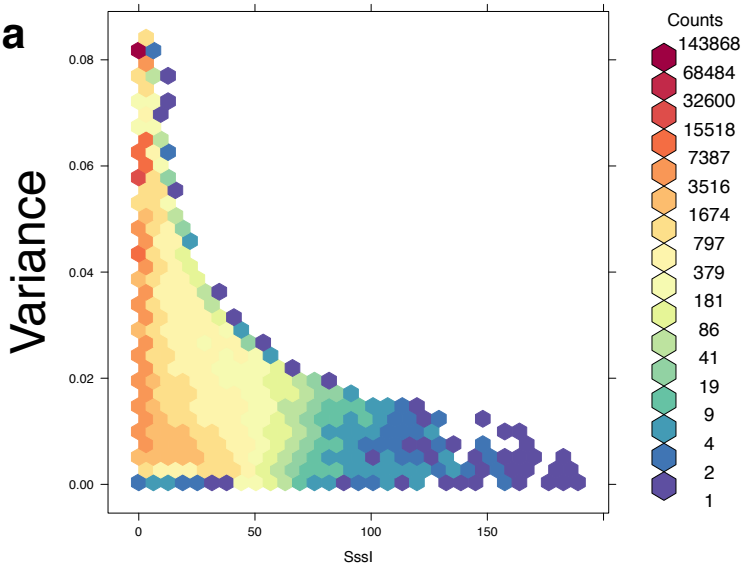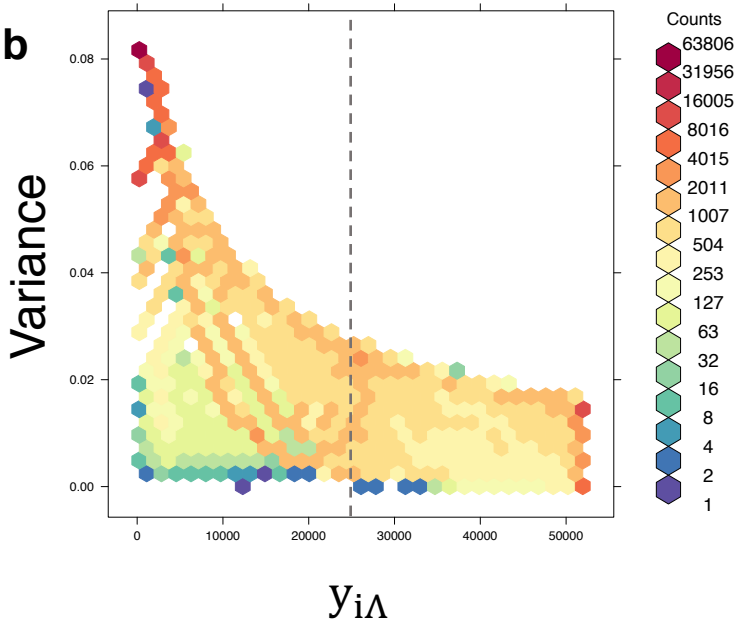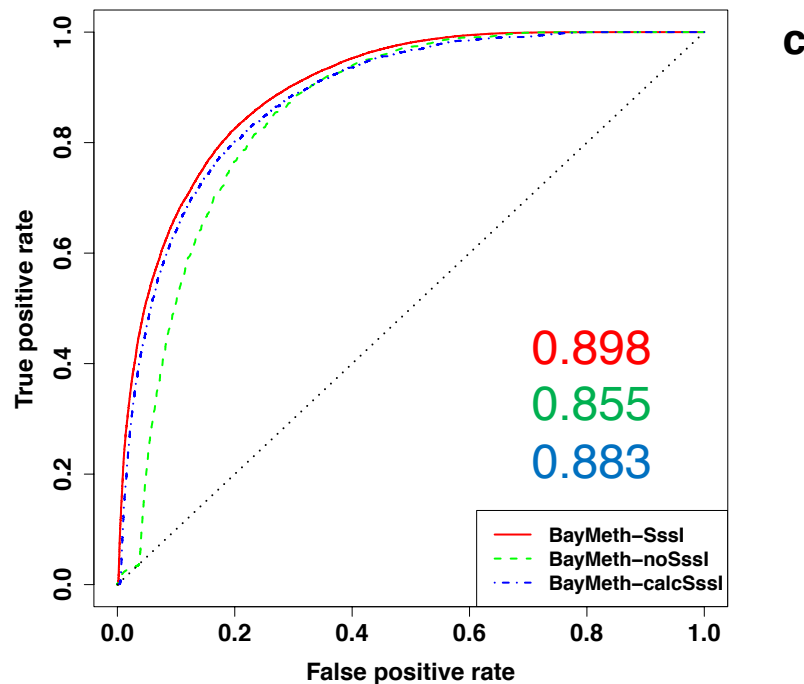

Supplement: Supplementary file 2 — Selecting a cutoff in yiΛ. Figure S1. Among windows in the Riebler et al. pulldown data set passing mappability and WGBS thresholds, we plot (a) the variance on the posterior probability of methylation from BayMeth-SssI against the measured SssI, which shows a similar decay profile but a difference in distribution from the plot of the (b) variance from BayMeth-calcSssI against yiΛ, our calculated SssI. In isolating a subset on which the methylation predictions can be more trusted when running BayMeth-calcSssI, we select a cutoff in yiΛ (dashed line) based on a region of stable variance on the prediction itself. The (c) ROC curves resulting from the methods being run on this subset with the AUC printed in the corresponding color. (PDF 245 kb) [file 12859_2019_3011_MOESM2_ESM.pdf]
